# New missense variants in *RELT* causing hypomineralised amelogenesis imperfecta

**DOI:** 10.1111/cge.13721

**Published:** 2020-02-21

**Authors:** Georgios Nikolopoulos, Claire E.L. Smith, Steven J. Brookes, Mohammed E. El‐Asrag, Catriona J. Brown, Anesha Patel, Gina Murillo, Mary J. O'Connell, Chris F. Inglehearn, Alan J. Mighell

**Affiliations:** ^1^ Division of Molecular Medicine, Leeds Institute of Medical Research The University of Leeds Leeds UK; ^2^ Department of Oral Biology, School of Dentistry, St James's University Hospital The University of Leeds Leeds UK; ^3^ Division of Cardiovascular Sciences, School of Medicine, Faculty of Biology Medicine and Health University of Manchester Manchester UK; ^4^ Department of Zoology, Faculty of Science Benha University Benha Egypt; ^5^ Birmingham Dental Hospital, Mill Pool Way, Edgbaston Birmingham UK; ^6^ School of Dentistry, Ciudad Universitaria Rodrigo Facio, Montes De Oca Universidad de Costa Rica San Jose Costa Rica; ^7^ School of Biology, Faculty of Biological Sciences The University of Leeds Leeds UK; ^8^ School of Life Sciences, Faculty of Medicine and Health Sciences The University of Nottingham Nottingham UK

**Keywords:** amelogenesis imperfecta, enamel, RELT, tumour necrosis factor receptor

## Abstract

Amelogenesis imperfecta (AI) is a heterogeneous group of genetic diseases characterised by dental enamel malformation. Pathogenic variants in at least 33 genes cause syndromic or non‐syndromic AI. Recently variants in *RELT*, encoding an orphan receptor in the tumour necrosis factor (TNF) superfamily, were found to cause recessive AI, as part of a syndrome encompassing small stature and severe childhood infections. Here we describe four additional families with autosomal recessive hypomineralised AI due to previously unreported homozygous mutations in *RELT*. Three families carried a homozygous missense variant in the fourth exon (c.164C>T, p.(T55I)) and a fourth family carried a homozygous missense variant in the 11th exon (c.1264C>T, p.(R422W)). We found no evidence of additional syndromic symptoms in affected individuals. Analyses of tooth microstructure with computerised tomography and scanning electron microscopy suggest a role for RELT in ameloblasts' coordination and interaction with the enamel matrix. Microsatellite genotyping in families segregating the T55I variant reveals a shared founder haplotype. These findings extend the *RELT* pathogenic variant spectrum, reveal a founder mutation in the UK Pakistani population and provide detailed analysis of human teeth affected by this hypomineralised phenotype, but do not support a possible syndromic presentation in all those with *RELT*‐variant associated AI.

## Graphical abstract

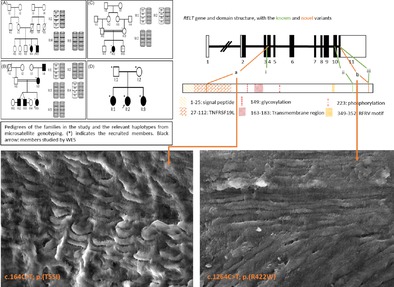

## 1 INTRODUCTION

Amelogenesis is the process by which dental enamel is formed during tooth development. It is characterised by three stages.^1^ During the initial secretory phase, a protein‐rich extracellular matrix (ECM) is secreted in a highly organised fashion by a monolayer of specialised cells known as ameloblasts that retreat away from a pre‐formed dentine surface. There is then a transition phase when the immature enamel reaches its final thickness, ECM secretion stops, and ECM proteins begin to be degraded in a controlled way. This is followed by a maturation stage during which ameloblasts pump mineral ions into the ECM, forming hydroxyapatite crystals which are organised in an intricate decussating pattern to make up the enamel prisms (rods), while the ECM proteins continue to be removed to make room for crystal growth.

Defects in proteins involved in any of these stages and processes can cause amelogenesis imperfecta (AI). AI is the common endpoint for an assortment of heterogeneous genetic enamel defects, presenting with an isolated or syndromic phenotype and typically inherited as X‐linked, dominant or recessive conditions. Thus far, variants in 19 genes have been implicated in non‐syndromic AI.^2^ A further 19 genes are listed in OMIM as being associated with syndromic forms of AI, with 5 implicated in both non‐syndromic and syndromic disease. Prevalence varies between populations, with frequency estimates of between 1 in 14000^3^ and 1 in 700.^4^ AI has an adverse impact on affected individuals often starting in early childhood, with poor aesthetics and functional failure. Discoloured, weak enamel can quickly break down causing pain, infection and early tooth loss with subsequent malocclusion. This in turn leads to high levels of distress, social avoidance, discomfort, isolation and emotional problems.^5^

Recently Kim and co‐workers reported one missense and two nonsense variants in the gene encoding Receptor Expressed in Lymphoid Tissues (*RELT*, OMIM: 611211), causing hypoplastic AI.^6^ Affected individuals were also of short stature and their medical history suggested increased susceptibility to childhood infection, potentially implying a syndromic form of AI.^6^

By homology, the 430‐amino acid RELT protein appears to be a receptor in the tumour necrosis factor (TNF) superfamily, which comprises 19 ligands (TNFs) and 29 receptors (TNFRs).^7^ It consists of an extracellular region with two cysteine‐rich domains homologous to other TNFR superfamily members and a predicted N‐glycosylation site, a central transmembrane domain and a unique intracellular domain.^8^ However, RELT is an orphan TNFR which does not appear to bind to any of the 19 known TNF ligands.^7^

RELT has been shown to be abundant in haematopoietic tissues, where it is involved in the activation of the NF‐κB pathway, inducing cell apoptosis by binding with TRAF1 (TNF receptor‐associated factor 1).^8^ Interestingly, RELT is unique among the TNFRs, as it does not have the characteristic death domain found in the intracellular region of the other TNFRs and, additionally, it only binds to TRAF1 and none of the other TRAF molecules, suggesting a non‐canonical TNFR pathway of apoptosis.^8^ It was also reported that the function of RELT might be dependent on the co‐expression of its two homologues RELL1 and RELL2 (RELT‐like 1 and 2), which are shown to induce apoptosis in the absence of trimeric TNF ligands.^9^ More recently, RELT, along with RELL1 and RELL2, were shown to activate p38 MAPK induced apoptosis when overexpressed.^10^

Prior to the study by Kim and colleagues, no connection had been made between RELT and any pathogenic phenotype in human or mouse models. Their study implicates RELT in the secretory stage of amelogenesis. Here we report two additional novel missense variants in the *RELT* gene that cause autosomal recessive hypomineralised AI in four families, confirming that mutations in *RELT* cause AI. Our findings extend the observed mutation spectrum, reveal a founder mutation common to three UK Pakistani families and provide detailed analysis of human teeth affected by this hypomineralised phenotype, but suggest that *RELT* variants are not always associated with a broader, syndromic phenotype.

## 2 MATERIALS AND METHODS

### 2.1 Patient enrolment

Affected and unaffected individuals were recruited with informed consent and local ethical approval from families segregating recessively‐inherited AI, in accordance with the declaration of Helsinki with local ethical approval (REC 13/YH/0028). A full medical history was taken and a diagnosis of AI made after clinical examination. Saliva was sampled using the OG‐500 saliva DNA extraction kits (DNA Genotek, Kanata, ON, Canada) and DNA was extracted from 500 μL of the sample according to manufacturer's instructions. An adult L7 molar was donated from the proband of family 2 and a primary incisor was donated from the proband of family 4. Each affected tooth was analysed concurrently with an appropriate control tooth obtained from the Skeletal Tissues Research Tissue Bank (School of Dentistry, University of Leeds; NRES REC 07/H1306/95+5).

### 2.2 Whole exome sequencing and analysis

**Figure 1:**
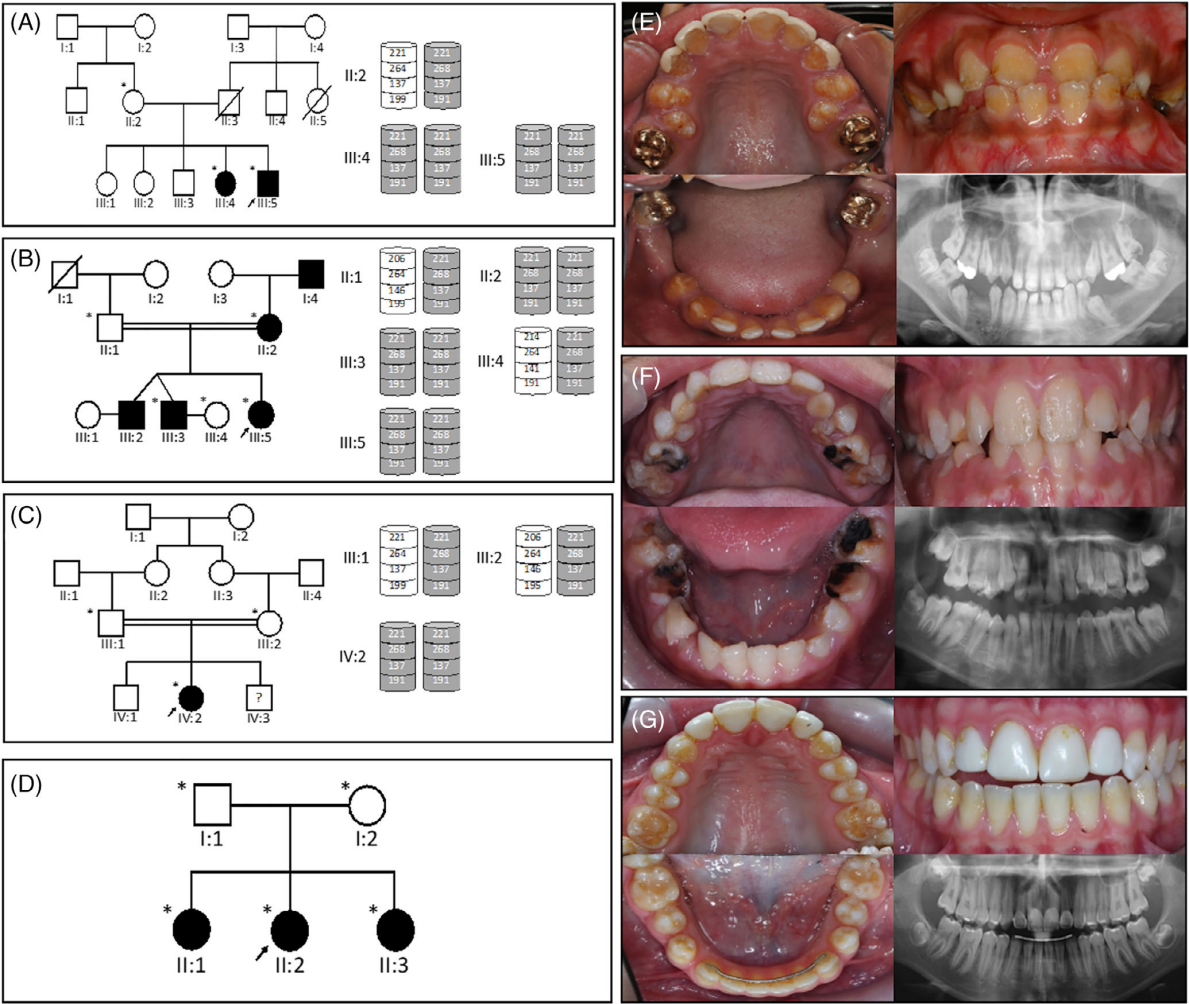
Four families with AI caused by homozygous variants in the *RELT* gene. Family 1 pedigree and haplotypes (A) and the clinical photos of the proband, III:5 (e). Family 2 pedigree and haplotypes (B), no clinical photos are available. Family 3 pedigree and haplotypes (C) and the clinical photos of the proband, IV:2 (F). Families 1‐3 carry the c.164C>T; p.(T55I) variant and share the same haplotype, marked grey. Family 4 pedigree (D) and clinical photos of the proband, II:2 (G), carrying the c.1264C>T; p.(R422W) variant. The recruited family members are marked with an asterisk and the proband in each family is indicated with a black arrow. There is variable, but mild, taurodontism involving permanent molar teeth, that is most obvious in the second molar teeth of family 4 [Colour figure can be viewed at http://wileyonlinelibrary.com]

Three micrograms of genomic DNA, from the proband in each family, marked with an arrow on the respective pedigree (Figure 1A‐D), were subjected to whole exome sequencing (WES). Exome capture was performed using the Agilent SureSelect Human All Exon Enrichment System. Libraries were sequenced with a 150 bp paired‐end protocol on an Illumina Hi‐Seq 3000 sequencer. Sequences were aligned and variants filtered as described previously.^11^

### 2.3 Sanger sequencing and segregation

Potentially pathogenic variants identified by WES were confirmed by PCR and Sanger sequencing, with segregation with disease confirmed for all available family members. The PCR primers used are listed in Table S1. Sequencing reactions were performed using the BigDye Terminator v3.1 kit (Life Technologies, California), according to the manufacturer's instructions, and sequences were resolved on an ABI3130xl sequencer (Life Technologies). Sequence analysis was carried out using the SeqScape v2.5 suite (Life Technologies).

### 2.4 Microsatellite analysis

Primer sequences for microsatellite markers were obtained from the UCSC genome browser and synthesised with 5′‐HEX‐tags (Table S1). PCR products were resolved on an ABI3130xl sequencer (Life Technologies) and sized relative to GeneScan 500 ROX (Life Technologies). Results were analysed using Genemapper v4.0 (Life Technologies).

### 2.5 Protein tertiary structure prediction

The sequence of the human RELT protein was retrieved from Uniprot (https://www.uniprot.org/uniprot/Q969Z4) and a homology model was produced in silico, by selecting the structure with the lowest energy conformation and then performing Molecular Dynamics simulations to examine the effects of the variants on the structure. Full methods used are detailed in Supporting Information section.

### 2.6 Tooth phenotyping: computerised X‐ray tomography

Teeth were obtained either after extraction for clinical reasons or as naturally shed primary teeth, from individuals with and without AI. These were analysed by micro‐computerised X‐ray tomography (μCT) using a Skyscan 1172 scanner (Bruker, Billerica, Massachusetts) operated at 100 kV with a source current of 100 μA. Two aluminium filters were used to prevent beam hardening artefacts. μCT images were reconstructed with Skyscan Recon software (Bruker). Hydroxyapatite mineral, of known densities (0.25, 0.75, and 2.9 g/cm^3^ (Bruker)), was used to calibrate the images. Calibrated false colour maps of mineral density were generated from the calibrated μCT images using ImageJ^12^ and the interactive 3D surface plot plugin (https://imagej.nih.gov/ij/plugins/surface-plot-3d.html). Videos were produced using CTVox software (Bruker).

### 2.7 Tooth sectioning and SEM

Using an Accutom‐5 cutter (Struers, Ballerup, Denmark) with a peripheral diamond cutting disc, cooled with minimal water, each tooth was cut across the bucco‐lingual axis. After sectioning, the cut edge was polished using 600 and 2000 grade carborundum paper (3M, Maplewood, Minnesota), followed by a nail buffer. Sections were etched by immersion for 20 seconds in 30% phosphoric acid and then washed with excess distilled water. The sections were dried under vacuum overnight, mounted on aluminium stubs and sputter coated with gold using an auto sputter coater (Agar Scientific, Elektron Technology, Stansted, UK). Microstructural analysis was performed using secondary electron detection in a Hitachi S‐3400 N scanning electron microscope (Hitachi, Tokyo, Japan), fitted with a 123 eV Nano XFlash Detector 5010 (Bruker) and operated at an accelerating voltage of 20 kV.

## 3 RESULTS

Four unrelated families with individuals presenting with AI were screened by WES to determine the genetic basis of their disease. These were part of a large cohort of AI families archived by the Leeds Dental Genetics Group. This cohort includes patients of various ethnicities with all three typical modes of inheritance, and over‐represents recessive disease due to high consanguinity in the local Pakistani population.^13^

Family 1 is a UK Pakistani family with the proband and one sibling presenting with hypomineralised AI (Figure 1A,E). Radiographs of unerupted permanent teeth show an apparently normal enamel volume with a difference in radiodensity between enamel and dentine, with loss of the normal crown contours after eruption (Figure S1A). Teeth in the mouth have variable loss of enamel consistent with post‐eruptive changes characterised by irregular surface loss and associated discolouration that progressed over time. The other three siblings and both parents are unaffected and there is no family history of AI reported. The parents of the proband reported that they were thought to be distant relatives, suggesting potential consanguinity. The medical histories of the affected children lack any reference to recurrent infection during infancy and there are no other recognised co‐segregating clinical features.

WES analysis of the proband revealed a *RELT* missense variant that segregates with disease in all family members in an autosomal recessive manner. Affected individuals are homozygous for a variant in *RELT* exon 4: c.164C>T [Refseq: NM_032871.3], p.(T55I) [NP_116260.2]. This variant is not present in gnomAD or EVS and is predicted to be pathogenic by both MutationTaster2 (Disease causing, p: 0.951) and CADD v1.3 (score: 27.6).

Family 2 is a consanguineous UK family, also of Pakistani origin (Figure 1B). The proband, her two brothers, mother and maternal grandfather are all reported to be affected. The radiographic phenotype was consistent with family 1 with no clinical images available (Figure S1B). No other potential syndromic features were apparent and there was no report of recurrent infections in infancy or childhood. WES analysis of the proband identified the same homozygous c.164C>T, p.(T55I) variant as in family 1.

Family 3 is also a consanguineous UK Pakistani family. The proband is the only one of three siblings diagnosed with AI, characterised by enamel surface irregularities and evidence of good enamel volumes on radiographs (Figure 1C,F; Figure S1C). Again, no other potentially syndromic features were noted and there was no history of recurrent infection in infancy. However, the proband and both other siblings were also diagnosed with isovaleric acidemia (IVA; OMIM #243500), an autosomal recessive condition in which the body is unable to metabolise leucine. IVA is caused by variants in the gene encoding isovaleryl‐CoA dehydrogenase (*IVD*) and is inherited in an autosomal dominant manner^14^ without any recognised impact on enamel formation. A pathogenic *IVD* variant had previously been identified in this family as part of clinical care. WES analysis of the proband revealed the same variant in exon 4 of *RELT* as in families 1 and 2, c.164C>T, p.(T55I), as well as the *IVD* variant.

Family 4 is a non‐consanguineous Costa Rican family presenting with AI in three children characterised by irregular surface loss of enamel and dental radiographs that included good enamel volumes (Figure 1D, G; Figure S1D). The parents do not have the same phenotype, although the mother presents with minor enamel irregularities on the cusps of the molars and canines. There is no report of any extraoral phenotype or recurrent infections in the family medical history.

WES analysis in the proband of family 4 identified a homozygous *RELT* variant in exon 11: c.1264C>T, p.(R422W), which segregated with the AI phenotype. This variant is in dbSNP (rs139368769) and is also present in gnomAD with MAF: 0.001633 and in EVS with MAF: 0.00077. It is predicted to be damaging by both MutationTaster2 (disease causing, p: 0.683) and CADD v1.3 (score: 34).

**Figure 2:**
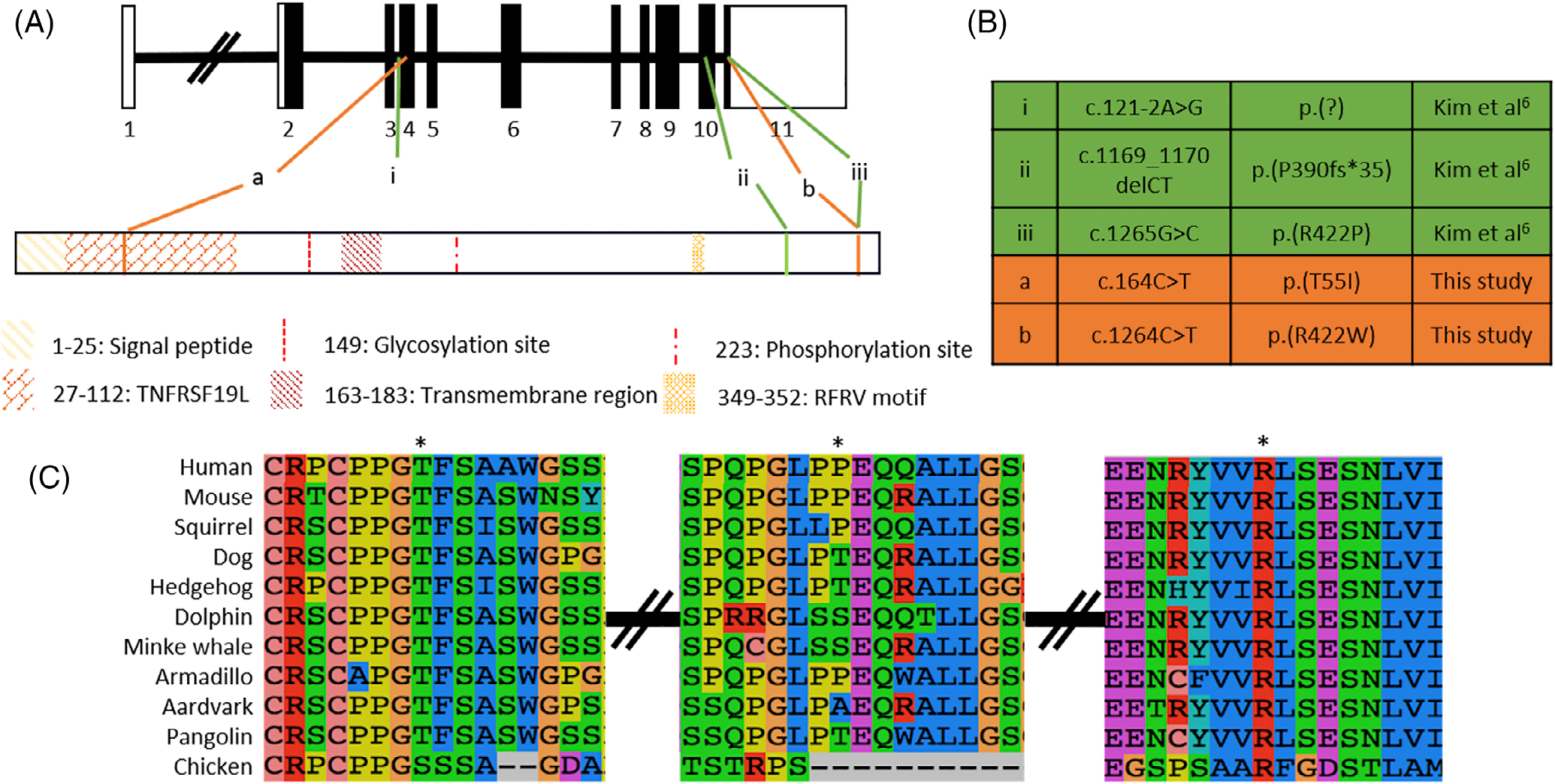
A, Gene diagram (NM_032871.3) and domain architecture (NP_116260.2) of *RELT*. B, Known pathogenic variants (green) and novel variants (orange). C, Conservation of the coding region variants [Colour figure can be viewed at http://wileyonlinelibrary.com]

The coverage statistics of the WES analysis of the probands of each family, including the mean coverage, are presented in Table S2. The electropherograms produced by Sanger sequencing and used for the segregation of each *RELT* variant are shown in Figures S2 and S3. Novel and published *RELT* variants and their position in the gene and on the protein are illustrated in Figure 2A and listed in detail in Figure 2B. Conservation of the variants on the coding sequence is shown at the amino acid level in Figure 2c, based on the sequences of Table S3.

As AI in families 1 to 3 results from homozygosity for the same variant and the families have the same ethnic background, we determined whether the affected family members share the same haplotype. Genotyping all available family members, from each family, with microsatellite markers flanking *RELT* (D11S916, D11S1314, D11S2371 and D11S4184) confirmed that they share a common haplotype, implying that the three families are distantly related (Figure 1A, B, C and Table S4).

**Figure 3:**
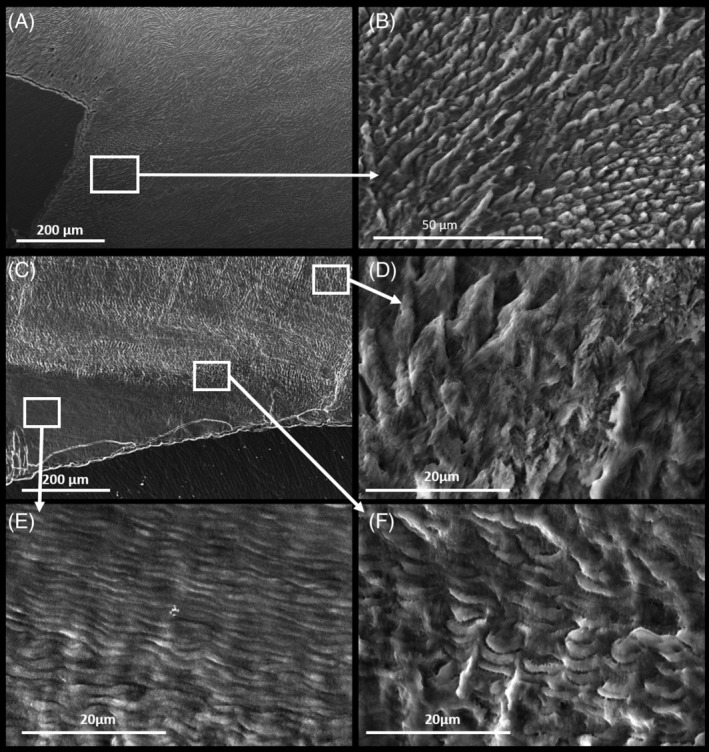
SEM photos of adult molars. A,B, Control L7 molar. C, Affected L7 molar from the proband (IV:2) of family 3, with the inserts showing the outer layer of enamel with normal looking prism organisation (D) that contrasts with abnormalities of the inner layer of stratified enamel (E) and the transitional phase (F) between the layers

An adult L7 molar from the proband (IV:2) of family 3 and a deciduous incisor from the proband (II:2) of family 4 were analysed with μCT and then scanning electron microscopy (SEM), with appropriate controls. A section of the μCT scan for each tooth has been false colour calibrated for hydroxyapatite density and is shown in Figure S4. Both control teeth display the expected prismatic enamel structure (Figure 3A,B for the molar and Figure S5A,B for the incisor). Analysis of the affected L7 molar revealed two layers of enamel (Figure 3C). The outer enamel layer has enamel prisms with abnormalities (Figure 3D), while the inner enamel layer is non‐prismatic with an abnormal lamellal structure (Figure 3E, F). The affected incisor of the proband (II:2) of Family 4 also has two distinctive layers of enamel (Figure S5C). The inner layer shows the regular configuration of enamel prisms which then become disorganised and change orientation to become non‐prismatic layered enamel (Figure S5D‐F). No enamel pits were identified. There is no indication in either tooth that the dentine or the dentine‐enamel junction are affected.

The tertiary structure of RELT was predicted using homology models to structures of other TNFRs that are available in PDB. The resulting model is presented in Figure S6. Domain location analysis identified four main regions: signal peptide (M1 to T225), Tumour Necrosis Factor Receptor (TNFR) (T27‐S112), Transmembrane domain (Y163 to C183) and RFRV motif (R349 to V352). Secondary structure prediction showed two α‐helices and sixteen β‐sheets for the protein. Site Directed Mutator (SDM) scores and the residue occluded surface packing density (OSP) calculated for the four variants show a consistent decrease in protein stability (Figure S6A, inserts).

## 4 DISCUSSION

In this study we report four families segregating autosomal recessive AI associated with two novel *RELT* variants. This confirms the finding of Kim and colleagues that mutations in *RELT* cause an AI phenotype,^6^ and extends the observed spectrum of pathogenic variants, bringing the total to five (http://dna2.leeds.ac.uk/LOVD/genes/RELT). These include a variant in the splice acceptor site at the end of intron 3 which disrupts normal splicing, a 2 bp deletion and a frameshift variant in exon 10, as well as missense variants in exons 4 and 11. The fact that two of the three missense variants alter residue R422 could potentially imply some unique function in tooth formation for this site in the protein, which is disrupted by these variants. However, the high pathogenicity prediction scores for these variants and the presence of likely null mutations, together with a recessive pattern of inheritance, instead suggest a haploinsufficiency mechanism, with complete loss of RELT function in affected individuals. Additional reported mutations will further illuminate the likely disease mechanism underlying AI due to variants in *RELT*.

The four families presented have a shared enamel phenotype with normal or near‐normal enamel volume present prior to tooth eruption. Post‐eruptive changes are rapid and lead to enamel loss, particularly at sites subject to physical loading such as occlusal surfaces. This phenotype shares features with that reported for *Relt* ^−/−^ mice, which have hypomineralised, full thickness enamel with a typical arrangement of enamel prisms.^6^ Although the difference in degree of mineralisation in AI and control teeth analysed in this study was modest, the overall features are most consistent with a hypomineralised form of AI. This highlights that “hypomineralised AI” is poorly defined and that the term does not take account of any variations in how the hydroxyapatite mineral is organised to give the resistance to physical attrition that characterises normal enamel. The enamel phenotype described here contrasts with the more severe phenotype of diminished enamel matrix formation leading to hypoplastic AI reported previously for *RELT* variants.^6^ Additionally, there is variable but mild taurodontism involving permanent molar teeth, identified in the lower right second molar of family 3 (Figure S1) and the second molar of family 4 (Figure 1G) and consistent with the radiographs for family 3 reported by Kim and colleagues.^6^ This radiographic feature is not as dramatic as that associated with AI due to *DLX3* genetic variants. The evaluation of future *RELT* cases will clarify whether taurodontism is a consistent feature and whether this has any implications for clinical care, as well as help to determine the spectrum of enamel phenotypes due to *RELT* variations.

Early enamel attrition after eruption is probably a consequence of abnormal enamel structure even though enamel prisms are present. The disorganised, non‐prismatic layers of enamel noted in Figure 3 and Figure S5 resemble closely the enamel architecture in AI patients carrying heterozygous *LAMB3* variants, as reported by Smith et al.^11^ AI‐causing variants in *LAMB3* and other hemidesmosomal proteins are thought to result in defective attachment of ameloblasts to the enamel matrix. Teeth from families with AI due to either *LAMB3* or *RELT* variants have layers of disorganised, non‐prismatic enamel lamellae and areas where enamel is prismatic but abnormal. These similarities of expression staging and phenotype could suggest a possible common mechanism for these different forms of AI. Of note is the recent report that ADAM10, a protein known to release anchored proteins, is expressed during the secretory phase of amelogenesis (but not thereafter) and that ADAM 10 cleaves the extracellular domain of RELT.^15^ Lamellal structures were not present in murine *Relt* ^−/−^ enamel, although there was an absence of the poorly mineralised spaces that would normally be expected at the dentino‐enamel junction.

Kim and colleagues reported that both heterozygous parents of family 1, carrying the c.1169_1170delCT variant, presented with a mild AI phenotype, consisting of subtle, localised enamel surface roughness and vertical enamel layers.^6^ This carrier phenotype was not noted in the other two families presented. Heterozygous members of the families reported in this study show no enamel abnormalities or malformations, with the exception of the mother (I:2) in family 4, who shows lesions on the cusps of her molars and canines. These could constitute a mild carrier phenotype in I:2, but these findings are absent from the father (I:1), who is also a heterozygous carrier of the same variant. Further studies are therefore required to confirm or exclude the existence of a mild carrier phenotype for AI due to *RELT* variants.

The protein structure of RELT has not been experimentally observed, and its function and the specific ligand that binds to it have not yet been characterised. We attempted to predict the tertiary structure of RELT using homology recognition. The homology of *RELT* to existing models only spans the TNFR region which accounts for 26% of the protein sequence (Figure S6). Mutating the predicted structure shows a probable decrease in protein stability for all of the variants implicated in AI causation. However, given the limited homology to other available models, the *ab initio* prediction of the structure of the entire peptide should be interpreted with caution, as should the predicted effect that the pathogenic variants will have on protein structure.

The previous report on biallelic *RELT* variants causing AI included evidence suggestive of a syndromic phenotype, including short stature and recurrent infantile infections. Kim and colleagues noted conservation of the *RELT* sequence, even in species that have no teeth, suggesting that this provided further support for probably syndromic consequences. However, sequence conservation across species need not imply conserved function, since proteins can have multiple functions, functions can change through time or the primary function can become obsolete.^16^ Thus, proteins with an enamel‐specific function might also have other roles in animals that have lost their teeth, such as pangolin (*Manis javanica*), platypus (*Ornithorhynchus anatinus*) and the baleen whales, for example, minke whale (*Balaenoptera acutorostrata*), or that do not have enamel, such as aardvark (*Orycteropus afer*), two‐toed sloth (*Choloepus hoffmanni*) and other members of their families. For example, AMBN, another protein involved in the secretory stage of amelogenesis,^17^ has been shown to participate in cell adhesion and proliferation,^18^ and has roles in adipose cells^19^ and in osteogenesis in mice.^20^, ^21^ Nevertheless, *AMBN* mutations appear to cause only non‐syndromic AI. Examination of patients from the four families recruited in this study and of their medical histories gave no indication of additional features. Our findings therefore do not support a clear syndromic phenotype for all *RELT* variants. As further cases are reported the extent of the syndromic phenotype should become clearer.

In conclusion, we confirm that biallelic variants in *RELT* cause hypomineralised AI characterised by normal or near normal enamel volumes, but leading to enamel that is prone to wear rapidly due to attrition. We find no evidence to support a clear syndromic element in the families reported here. This study extends the observed pathogenic variant spectrum and reveals a common founder mutation in UK Pakistani AI families. We also show how human enamel structure is affected, with regions of both layered enamel and enamel which retains some prismatic structure but is abnormally formed. We note similarities to the phenotype seen in teeth of patients carrying *LAMB3* variants, indicating a possible connection between the role of RELT in amelogenesis and attachment of the ameloblasts to the enamel matrix.

## CONFLICT OF INTEREST

The authors declare no potential conflicts of interest with respect to the authorship and/or publication of this article.

## Supporting information


**Table S1**: Primers used for the PCR amplifications and the microsatellite analyses.
**Table S2**: Coverage statistics for the WES of families 1‐4
**Table S3**: RELT sequences used for conservation analysis
**Table S4**: Detailed results of the microsatellite analysis for families 1, 2 and 3
**Figure S1**: Radiographs from the four families with AI caused by homozygous variants in the *RELT* gene.
**Figure S2**: Electropherograms of families 1, 2 and 3
**Figure S3**: Electropherograms of family 4
**Figure S4**: Calibrated enamel density heatmaps of microCT scan sections
**Figure S5**: SEM photos of deciduous incisors
**Figure S6**: Structural modelling of the tumour necrosis factor receptor RELT.

## Data Availability

The variants reported in this study have been deposited to ClinVar with accession numbers SCV000998810 and SCV000998811 and to the AI Leiden Open Variation Database (LOVD ‐ https://dna2.leeds.ac.uk/LOVD/) with accession numbers RELT_000004 and RELT_000005, respectively.
