# Supplementary material for: New missense variants in RELT causing hypomineralised amelogenesis imperfecta
Source: Clin Genet. 2020 Feb 21;97(5):688–95. doi: 10.1111/cge.13721 (PMC7216828; doi:10.1111/cge.13721)
Supplement: Supplementary file 1 — Table S1: Primers used for the PCR amplifications and the microsatellite analyses. Table S2: Coverage statistics for the WES of families 1‐4 Table S3: RELT sequences used for conservation analysis Table S4: Detailed results of the microsatellite analysis for families 1, 2 and 3 Figure S1: Radiographs from the four families with AI caused by homozygous variants in the RELT gene. Figure S2: Electropherograms of families 1, 2 and 3 Figure S3: Electropherograms of family 4 Figure S4: Calibrated enamel density heatmaps of microCT scan sections Figure S5: SEM photos of deciduous incisors Figure S6: Structural modelling of the tumour necrosis factor receptor RELT. [file CGE-97-688-s001.docx]

**New missense variants in *RELT* causing hypomineralised amelogenesis imperfecta**

Georgios Nikolopoulos^1,2^, Claire E. L. Smith^1^, Steven J. Brookes^2^, Mohammed E. El-Asrag^1,3,4^, Catriona J. Brown^5^, Anesha Patel^5^, Gina Murillo^6^, Mary J. O’Connell^7,8^, Chris F. Inglehearn^1,9^ and Alan J. Mighell^2,9*^

**Supplemental Data Contents**

**Supplemental Tables**

Table S1: Primers used for the PCR amplifications and the microsatellite analyses.

Table S2: Coverage statistics for the WES of Families 1-4

Table S3: RELT sequences used for conservation analysis

Table S4: Detailed results of the microsatellite analysis for families 1, 2 and 3

| **Target** | **Forward primer** | **Primer Tm (˚C)** | **Reverse primer** | **Primer Tm (˚C)** | **Expected product size (bp)** |
| --- | --- | --- | --- | --- | --- |
| *RELT*_exon4 | CGACCTGGTGAGCATTGC | 59 | GCCAGCAGTCTCCACAGA | 60 | 299 |
| *RELT*_exon11 | AAGGAGAAAGGCATCTGTTGG | 57 | CTTCTCGGTCCTCACAGTCC | 60 | 297 |
| D11S1314 | HEX-TTGCTACGCACTCCTCTACT | 57 | GTGAAGGCAGGAAATGTGAC | 56 | 209-227 |
| D11S4184 | HEX-CCCAGCCTTACATATTCC | 52 | GCTGATGAGCAGAGGTAG | 54 | 263-277 |
| D11S916 | HEX-CAGACTATTCTCATTGCTGC | 53 | GGACTTCTAAGCCTCCATAA | 53 | 135-153 |
| D11S2371 | HEX-CTGAGGTGGGAGGTTCAGTT | 59 | CCCGGCCTTGATTTATTTAA | 52 | 193-213 |

**Table S1**: Primers used for the PCR amplifications and the microsatellite analyses.

| **Family** | **Family member** | **Mean Coverage** | **% Bases covered >4x** | **% Bases covered >14x** | **% Bases covered >19x** |
| --- | --- | --- | --- | --- | --- |
| 1 | III:5 | 68.69 | 98.9 | 96.4 | 94.0 |
| 2 | III:5 | 52.04 | 99.4 | 95.6 | 91.1 |
| 3 | IV:2 | 54.09 | 99.4 | 96.0 | 91.9 |
| 4 | II:2 | 54.70 | 99.4 | 96.2 | 92.5 |

**Table S2**: Coverage statistics for the WES of Families 1-4

| **Species** | **Accession No** | **Amino acid sequence** |
| --- | --- | --- |
| *Homo sapiens* (Human) | ENST00000064780 | MKPSLLCRPLSCFLMLLPWPLATLTSTTLWQCPPGEEPDLDPGQGTLCRPCPPGTFSAAWGSSPCQPHARCSLWRRLEAQVGMATRDTLCGDCWPGWFGPWGVPRVPCQPCSWAPLGTHGCDEWGRRARRGVEVAAGASSGGETRQPGNGTRAGGPEETAAQYAVIAIVPVFCLMGLLGILVCNLLKRKGYHCTAHKEVGPGPGGGGSGINPAYRTEDANEDTIGVLVRLITEKKENAAALEELLKEYHSKQLVQTSHRPVSKLPPAPPNVPHICPHRHHLHTVQGLASLSGPCCSRCSQKKWPEVLLSPEAVAATTPVPSLLPNPTRVPKAGAKAGRQGEITILSVGRFRVARIPEQRTSSMVSEVKTITEAGPSWGDLPDSPQPGLPPEQQALLGSGGSRTKWLKPPAENKAEENRYVVRLSESNLVI |
| *Mus musculus* (Mouse) | ENSMUST00000008462 | MKRTLLCWPLSCLFVLLPWPLATPTPITPWLCPPGKEPDPDPGQGTLCRTCPPGTFSASWNSYPCQPHYRCSLQKRLEAQAGTATHDTMCGDCQHGWFGPQGVPHVPCQPCSKAPPSTGGCDESGRRGRRGVEVAAGTSSNGEPRQPGNGTRAGGPEETAAQYAVIAIVPVFCLMGLLGILVCNLLKRKGYHCTAQKEVGPSPGGGGSGINPAYRTEDANEDTIGVLVRLITEKKENAAALEELLKEYHSKQLVQTSHRPVPRLLPASPSIPHICPHHHHLHTVQGLASLSGPCCSRCSQKWPEVLLSPEAAAATTPAPTLLPTASRAPKASAKPGRQGEITILSVGRFRVARIPEQRTSSLLSEVKTITEAGPSEGDLPDSPQPGLPPEQRALLGSGGSHTKWLKPPAENKAEENRYVVRLSESNLVI |
| *Ictidomys tridecemlineatus*  (Squirrel) | ENSSTOT00000014494 | MKLSGLCWPLSCLLVLLPWPLATATPTTLWQCPPGEEPDLDQGQGTLCRSCPPGTFSISWGSSPCQPHDRCSLRRRLEVQAGTATQDTLCGGCQPGWIGPRGVPHIPCQPCSWASLSTPGCNEWGRRARRGVEVAAGSSGGGETQQPGNGTRAGGPEETAAQYAVIAIVPIFCLMGLLGILVCNLLKRKGYHCTAHKEVGPGPGGGGSGINPAYRTEDANEDTIGVLVRLITEKKENAAALEELLKEYHSKQLVQTSHRPVPRLPPASPSMPHICPHRHHLHTVQGLASLSGPCCSRCSQKKWPEVLLSPEAAAATTPAPSLLPNPARAPKAGAKTGRQGEITILSVGRFRVARIPEQRTSSAASEVKTITEAGPSGGDLPDSPQPGLLPEQQALLGSGGSHTKWLKPPAENKAEENRYVVRLSESNLVI |
| *Canis lupus familiaris* (Dog) | ENSCAFT00000009030 | MKLSWPHWPLSCLFVLLPWPLATPISTTPWQCPPGEEPSLDLGQGTLCRSCPPGTFSASWGPGPCQPHSRCSPRGRLEAQPGTATQDTLCGDCQPGWFAASEVTHVPCQLCPWTPLGIRSCYERGRRARRGVEVAAGTTGTGDTRQPGNGTRAGGPEETAAQYAVIAIVPVFCLMGLLGILVCNLLKRKGYHCTAHKEVGPGPGGGGSGINPAYRAEDANEDTIGVLVRLITEKKENAAALEELLKEYHSKQLVQTSHRPVPRLPPGPPSMPHICPHRHHLHTVQGLASLSGPCCSRCSQKKWPEVLLSPEAAAATTPTPRVLPNLARAPKAGAKAGRQGEITILSVGRFRVARIPEQRSSSAASELKTITEAGPSGGDLPDSPQPGLPTEQRALLGSGGSHTKWLKPPAENKAEENRYVVRLSESNLVI |
| *Erinaceus europaeus*  (Hedgehog) | XM_016190165 | MKPSPEQLWPLSCLLVLLPSSLATPTLASWLCPPGEEPELHSAQDTFCRPCPPGTFSISWGSSPCQPHSRCNPRGRLEAQAGTATRDALCGDCRPGWFATPEAPQAPCRPPATHDLYGQRTRRGVEVAAAAGSSEKTQQLGNGTQAGSTEERAAQYAVIAIVPVFCLMGLLGILVCNLLKRKGYHCTAHKEVGPGPGGDGNGINPAYLAEDTNEDTIGVLVRLITEKKENAAALEELLKEYHSRQLAQTSHRPVPRLPLGSPRAPHTCPHGHHLHTVQGLASLSGPCCSRCSQKKWPEVLLSPEAAAATSPTPRLLTNPGRAPKAVPKAGRQGEITILSVGRFRVARIPEQRTSSAVSEVKTITEAGPVGGSLPDSPQPGLPTEQRALLGGRGSHTKWLKPPAEDKTEENHYVIRLSESNLVI |
| *Tursiops truncatus* (Dolphin) | ENSTTRT00000004627 | MKLSPPHWPLSCLLVLLPWPLATATSTTPWPCPPGEEPNLEPGRGTLCRSCPPGTFSASWGSSPCQPHSRCSPQGRLEAQVGTATRDTLCGNCQPGWFAPSEVPHVPCRPCSWTPLGTRGCYERGRRARRGMEVAARASGAGETRQPGNSTRAGSPEETAAQYAVIAIVPIFCLMGLLGILVCNLLKRKGYHCTAHKEVGPGPGGGGSGVNPAYHTDDANEDTIGVLVRLITEKKENAAALEELLKEYHSKQLVQTSHRPLPRLPPGPPSMPHVCPHRHHLHTVQGLASLSGPCCSRCSQKWPEVLLSPEAAAATTPTPRLLPNPARVPKVGAKAGRQGEITILSVGRFRVARIPEQRMSSVASEVKTITEAGPSGGDLPDSPRRGLSSEQQTLLGSGGSHPKWLKSPAENKAEENRYVVRLSESNLVI |
| *Balaenoptera acutorostrata*  (Minke whale) | XM_007173703 | MKLSPPHWPLSCLLVLLPWPLATATSTTPWPCPPGEEPNLEPGQGTLCRSCPPGTFSASWGSSPCQPHSRCSPRGRLEAQVGTATRDTLCGNCQPGWFAPSEVPRVPCQPCSWTPLGTRGCYERGRRARRGVEVAAGASGAGETRQPGNSTRAGSPEETAAQYAVIAIVPIFCLMGLLGILVCNLLKRKGYHCTAHKEVGPGPGGGGSGVNPAYQTEDTNEDTIGVLVRLITEKKENAAALEELLKEYHSKQLAQTSHRPLPRLPPGPPSIPHVCPHRHHLHTVQDLASLSGPCCSRCSQKWPEVLLSPEAAAATTPTPRLLPNPARAPKVGAKAGRQGEITILSVGRFRVARIPEQRTSSVASEVKTITEARPSGGDLPDSPQCGLSSEQRALLGSGGSHPKWLKPPAENKAEENRYVVRLSESNLVI |
| *Dasypus novemcinctus*  (Armadillo) | ENSDNOT00000042824 | MKLSPLRWPLSCLLVLLPWPPAAPTLAPPWPCPPGEEPHLDPGLGASCRSCAPGTFSASWGPGPCRPHARCGLRGRLEAQAGTATRDALCGGCQPGWFAPEGTLLAPCQLCSWAPPETRGCDAWGRRARRGVEVAAGAGGGRETRQPRNSTRAGGPEETAAQYAVIAIVPIFCLMGLLGILVCNLLKRKGYHCTAHKEVGPGPAGGGSGINLACRADEANEDTIGVLVRLITEKKENAAALEELLKEYHSKQLAQTSRRPVPRPPPGLPSVPHVCPHRHHLHTVQGLASLSGPCCSRCSQKKWPEVLLSPEAAAAAAPAPSLLPTLARAPKAGGKAGRQGEITILSVGRFRVARIPEQRTSEVKTITEAGPSGGDLPDSPQPGLPPEQWALLGSGGSHAKWPKPPAENKAEENCFVVRLSESNLVI |
| *Orycteropus afer*  (Aardvark) | XM_007941042 | MEALGEQDVASGLAESCAGTRSLQGPLLPWSPATPASITPWQCPPGEEPSLDLRQGELCRSCPPGTFSASWGPSPCQPHASCSPRGRLEAQVGSATRDTLCGGCWPGWFAPEGGPHVPCQPCSWAPLGTHGCDVWRHRVRRGVEASAGAGSSGETRQPGNSTRAGSPEETAAQYAVIAIVPVFCLMGLLGILVCNLLKRKGYHCTAHKEVGPDPGGGSSGNNPAYRAEDANEDTIGVLVRLITEKKENAAALEELLKEYHSKQLVQTNLRPVPRLPPGSSSMPHICPHRHHLHTVQGLASLSGPCCSRCSQKKWPEVLLSPEAAAATTPTPSFLPGSARVPKAGAKAGRQGEITILSVGRFRVARIPEQRTSSLASELKTITEAGPLGDDFPDSSQPGLPAEQRALLGSGTNHTKRLKHPTENKTEETRYVVRLSESNLVI |
| *Manis javanica*  (Pangolin) | XM_017646709 | MKLSLQHWPLSCLLVLLPWPLVTPTSTTPWQCPPGEEPNQDLGQGTSCRSCPPGTFSASWGSSPCQPHSHCRPQGRLEAQAGTATQDTLCGDCQSGWFAPSEAPHVPCRPCSWTPLRILSCYERGRRARRGVEVATGTSRAGETRQPGNSTRAGGPEETAAQYAVIAIVPVFCLMGLLGILVCNLLKQKGYHCTAHKEAGPGPGGGGSGINPAYRAEDANEDTIGVLVRLITEKKENAAALEELLKEYHSKQLVQTSHGPVPRLPLGSPNMLHICPHRYHLHTVQGLASVSGPCCSRCSQKKWPEVLLSPEAAAATTPTPRVLPNPARVPKTGAKAGCQGEITILSVGRFHVARIPEQRTSSMASEVKTVMEAGLSGGDLPDSSQPGLPTEQWALLGSGGSHTKWLNPPAENMAEENCYVVRLSESNLVI |
| *Gallus gallus*  (Chicken) | ENSGALT00000092464 | MRWWLLAVLGVLSSPCMGTTACELLRCPPGEEPIGACSTAQSCRPCPPGSSSAGDAPCACMHGFYSPDGHREPQGRCLPCSTAPHGTLGCAARRRARSVVAARGPSGTNGTWELQPEEAATAQSAVLAIVPVFCAMGLLGILVCNLLKKKGYHCTAHKEHEHSTSGPSSIYQIEDANEDTIGVLVRLITEKKENAAALEELLKEHQSMQPALAGCKPAYKLHLLPQFPQSCCHQQHLHTVHGPAPPSDPPCTRCSQRKWPQVLPSPTATKATRPAGEITILSIGRFRVSRIPEQKPGVGGDPLPTSTRPSWLKSTDSRPEGSPSAARFGDSTLAM |

**Table S3:** RELT sequences used for conservation analysis

|  |  | **D11S1314** | | **D11S4184** | | **D11S916** | | **D11S2371** | | **Affected** |
| --- | --- | --- | --- | --- | --- | --- | --- | --- | --- | --- |
| **Family** | **Family member** | **Allele A** | **Allele B** | **Allele A** | **Allele B** | **Allele A** | **Allele B** | **Allele A** | **Allele B** | **-** |
| 1 | II:2 | 221 | 221 | 264 | 268 | 135 | 137 | 191 | 199 | NO |
|  | III:4 | 221 | 221 | 268 | 268 | 137 | 137 | 191 | 191 | YES |
|  | III:5 | 221 | 221 | 268 | 268 | 137 | 137 | 191 | 191 | YES |
| 2 | II:1 | 206 | 221 | 264 | 268 | 137 | 146 | 191 | 199 | NO |
|  | III:4 | 214 | 221 | 264 | 268 | 137 | 141 | 191 | 191 | NO |
|  | II:2 | 221 | 221 | 268 | 268 | 137 | 137 | 191 | 191 | YES |
|  | III:3 | 221 | 221 | 268 | 268 | 137 | 137 | 191 | 191 | YES |
|  | III:5 | 221 | 221 | 268 | 268 | 137 | 137 | 191 | 191 | YES |
| 3 | III:1 | 221 | 225 | 264 | 268 | 133 | 137 | 191 | 199 | NO |
|  | III:2 | 206 | 221 | 268 | 268 | 137 | 146 | 191 | 195 | NO |
|  | IV:2 | 221 | 221 | 268 | 268 | 137 | 137 | 191 | 191 | YES |

**Table S4**: Detailed results of the microsatellite analysis for families 1, 2 and 3

**Supplemental Methods**

*Whole Exome Sequencing (WES) and analysis*

Sequences were aligned to the human reference genome (GRCh37) with the Burrows Wheeler Aligner^1^. Alignments were processed with SAMtools^2^, Picard tools (<http://picard.sourceforge.net>) and the Genome Analysis Toolkit (GATK v3.5)^3^ to correct indel sites and mark PCR duplicates. Biallelic variants were called using the Haplotype Caller of GATK and saved in the VCF format. The minor allele frequency (MAF) of the variants was retrieved from the Exome Variant Server (EVS, <https://evs.gs.washington.edu/EVS/>), gnomAD (<https://gnomad.broadinstitute.org/>)^4^ and dbSNP150^5^ and common variants (MAF ≥ 1%) were excluded. The likely pathogenicity of candidate variants was estimated using the MutationTaster2 (<http://www.mutationtaster.org>)^6^ and Combined Annotation Dependent Depletion (CADD v1.3, <https://cadd.gs.washington.edu>)^7^ prediction algorithms.

*Protein tertiary structure prediction*

The sequence of the human RELT protein in FASTA format was retrieved from UniProt (https://www.uniprot.org/uniprot/Q969Z4). A homology model of human wild type RELT was produced using MODELLER (https://salilab.org/modeller/)^8^. Sequence homology between the template and reference structure, obtained from the protein databank (PDB, https://www.rcsb.org)^9^, was calculated using BLAST and T-Coffee. The resulting models were scored using MolProbity (http://molprobity.biochem.duke.edu) and prioritized based on global quality estimate, sequence identity and coverage^10^. Structures of the wild type (T55 and R422) and the mutated (I55 and W422) proteins were created in UCSF Chimera^11^ and the lowest energy residue conformation was selected. Mutations were visualised on the model structure using VMD v1.9.3^12^ and colocalization of the functional domains were determined using PyMOL v2.3^13^. Coarse-grain structural analysis was carried out using the site directed mutator (SDM, <http://marid.bioc.cam>.ac.uk/sdm2/). The Molecular Dynamics simulations of the RELT wildtype and mutants were performed using Amber16 and calculation of implicit solvent effects in the molecular dynamics was carried out using the LCPO model^14^.

**Supplemental Figures**

Figure S1: Radiographs from the four families with AI caused by homozygous variants in the *RELT* gene.

Figure S2: Electropherograms of families 1, 2 and 3

Figure S3: Electropherograms of family 4

Figure S4: Calibrated enamel density heatmaps of microCT scan sections

Figure S5: SEM photos of deciduous incisors

Figure S6: Structural modelling of the tumor necrosis factor receptor RELT


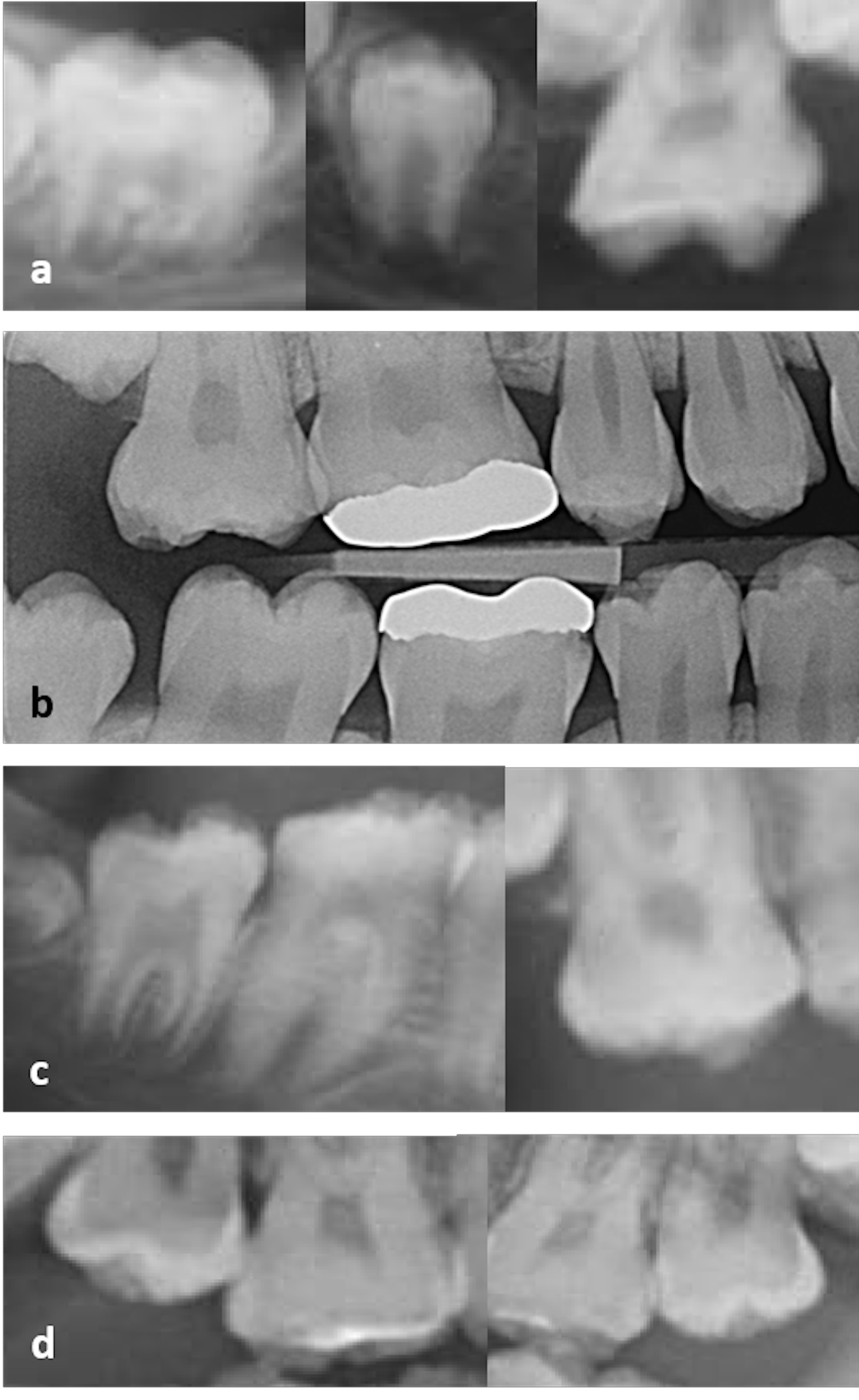


**Figure S1:** **Radiographs from the probands of the four families with AI caused by homozygous variants in the *RELT* gene.** a: Family 1, III:5. b: Family 2, III:5. c: Family 3, IV:2. d: Family 4: II:2. Collectively, the radiographs indicate a normal or near-normal enamel volume at the time of formation with a clear difference in radiodensity between enamel and dentine. Once teeth come into function, there is rapid loss of enamel at sites of physical loading such as occlusal surfaces, with maintenance of normal enamel contours at other sites. There is variable, but mild, taurodontism involving permanent molar teeth, that is most obvious in the lower right second molar of family 3. There was nothing on any of the radiographs to indicate developmental enamel hypoplasia.


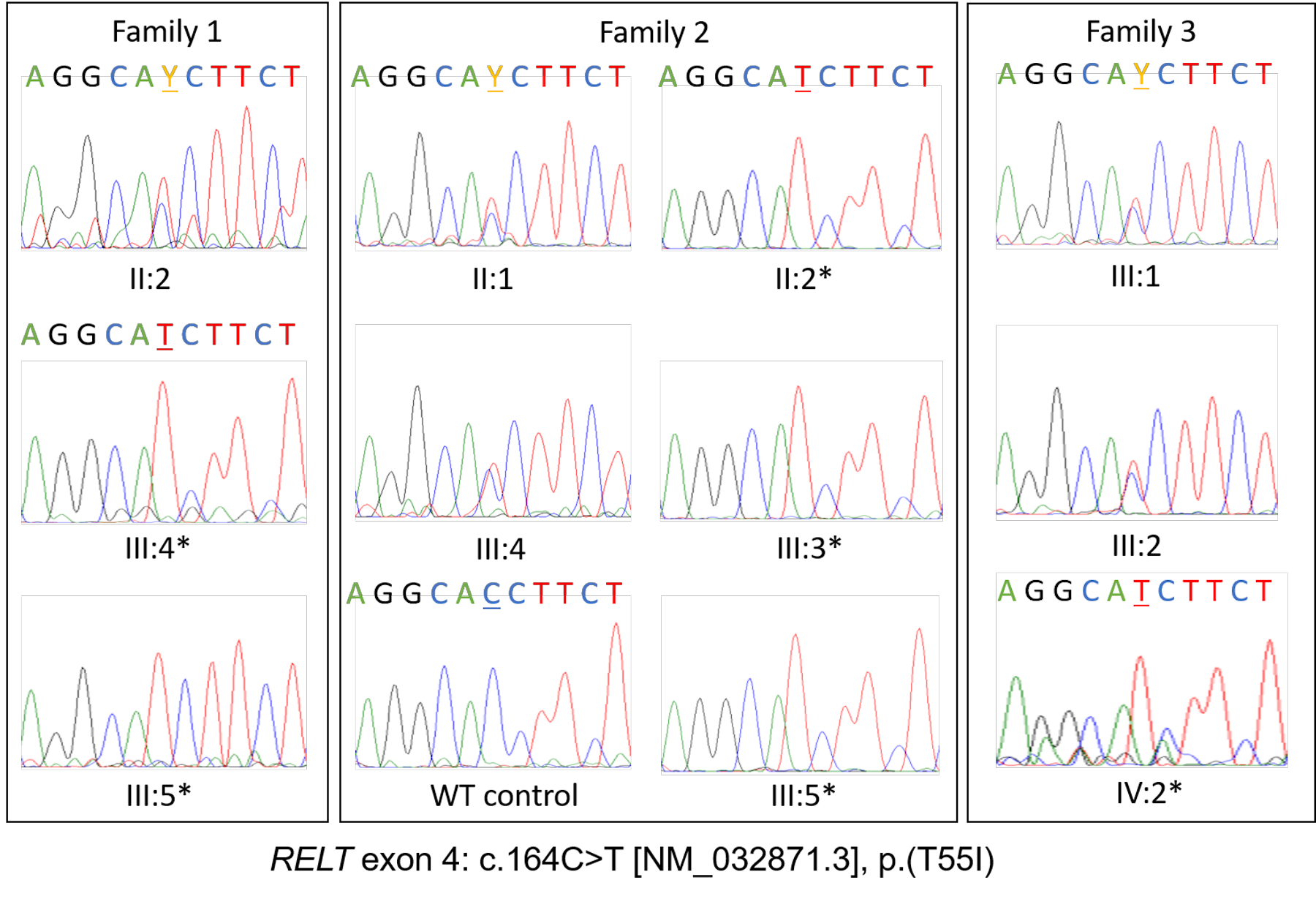


**Figure S2:** Electropherograms of recruited family members of families 1, 2 and 3. The affected individuals are indicated with an asterisk (*).


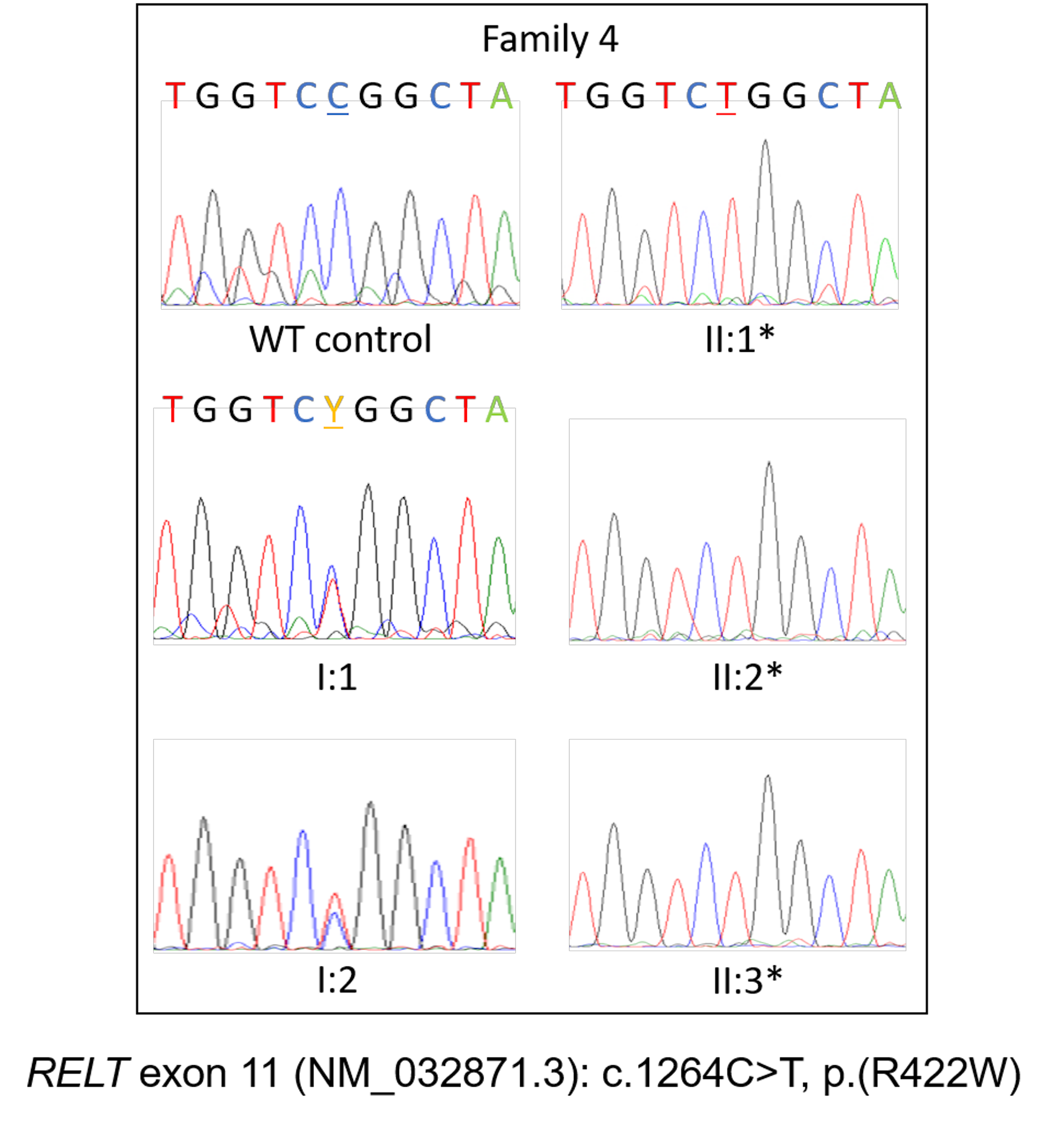


**Figure S3:** Electropherograms of recruited family members of family 4. The affected individuals are indicated with an asterisk (*).


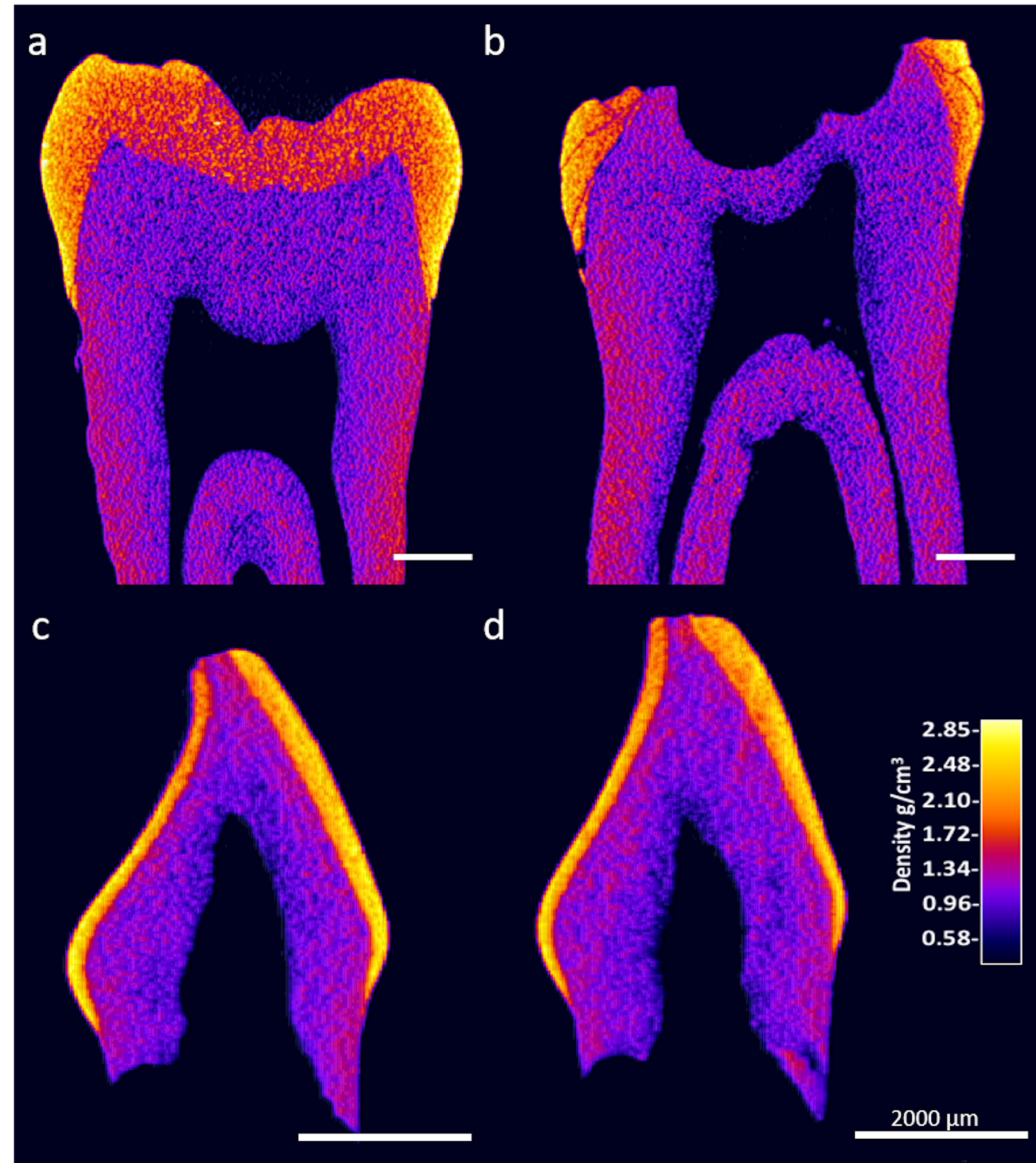


**Figure S4:** **Calibrated enamel density heatmaps of microCT scan sections.** A: Control L7 molar. B: affected L7 molar from family 2. The thickness of the enamel looks normal, but judgement is impeded by the corrosion of the crown. C: control deciduous incisor. D: affected deciduous incisor from family 4. The enamel thickness looks comparable between the AI and control teeth, which is consistent with a hypomineralised rather than a hypoplastic phenotype.


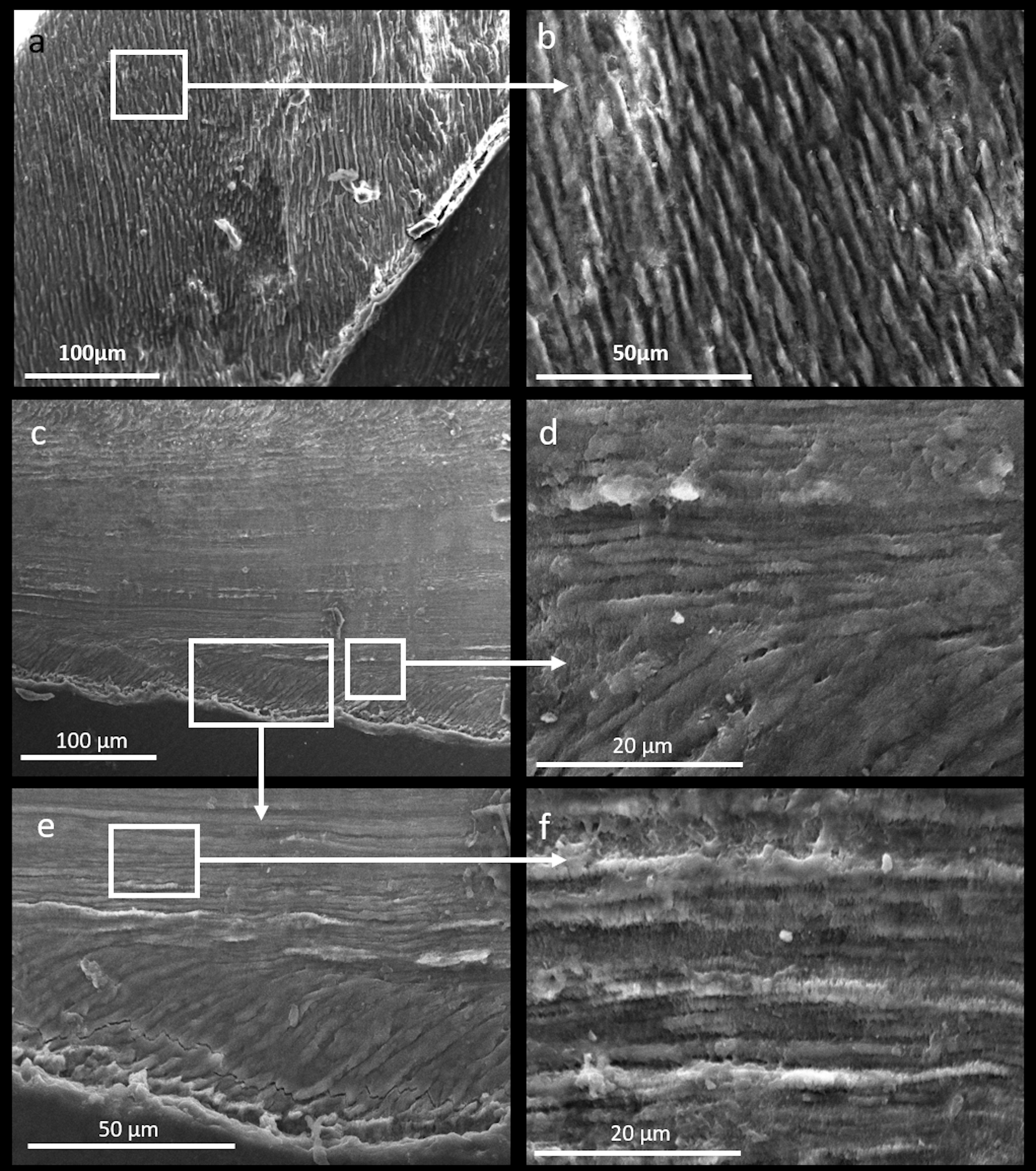


**Figure S5:** **SEM photos of deciduous incisors.** a, b: Control incisor. c: Affected incisor from the proband (II:2) of family 4, with the inserts showing the stratified enamel (d, e, f)


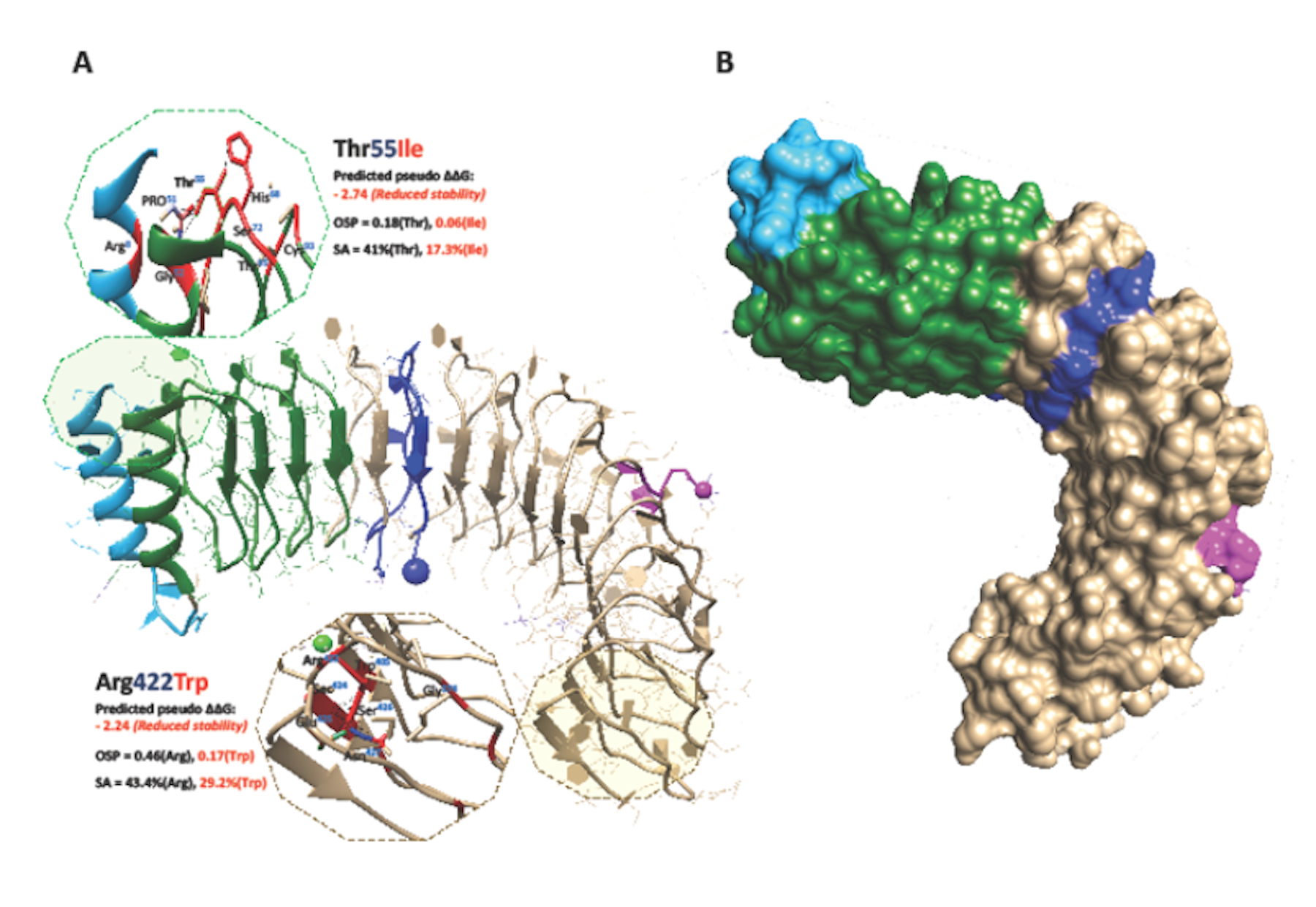


**Figure S6:** **Structural modelling of the tumor necrosis factor receptor RELT.** The signal peptide has been coloured light blue, the TNFR motif green, the transmembrane domain dark blue and the RFRV motif purple. A: The novel variants reported in this study, p.(T55I) and p.(R422W), are shown in the inserts and are predicted to decrease the stability of the peptide, indicated by the ΔΔG. B: Surface representation of the predicted RELT structure.

References

1. Li H, Durbin R. Fast and accurate short read alignment with Burrows-Wheeler transform. *Bioinformatics*. 2009;25(14):1754-1760. doi:10.1093/bioinformatics/btp324.

2. Li H, Handsaker B, Wysoker A, Fennell T, Ruan J, Homer N, Marth G, Abecasis G, Durbin R. The Sequence Alignment/Map format and SAMtools. *Bioinformatics*. 2009;25(16):2078-2079. doi:10.1093/bioinformatics/btp352.

3. Depristo MA, Banks E, Poplin R, Garimella K V., Maguire JR, Hartl C, Philippakis AA, Del Angel G, Rivas MA, Hanna M, McKenna A, Fennell TJ, Kernytsky AM, Sivachenko AY, Cibulskis K, Gabriel SB, Altshuler D, Daly MJ. A framework for variation discovery and genotyping using next-generation DNA sequencing data. *Nat Genet*. 2011;43(5):491-501. doi:10.1038/ng.806.

4. Karczewski KJ, Francioli LC, Tiao G, Cummings BB, Alföldi J, Wang Q, Collins RL, Laricchia KM, Ganna A, Birnbaum DP, Gauthier LD, Brand H, Solomonson M, Watts NA, Rhodes D, Singer-Berk M, Seaby EG, Kosmicki JA, Walters RK, Tashman K, Farjoun Y, Banks E, Poterba T, Wang A, Seed C, Whiffin N, Chong JX, Samocha KE, Pierce-Hoffman E, Zappala Z, O’Donnell-Luria AH, Minikel EV, Weisburd B, Lek M, Ware JS, Vittal C, Armean IM, Bergelson L, Cibulskis K, Connolly KM, Covarrubias M, Donnelly S, Ferriera S, Gabriel S, Gentry J, Gupta N, Jeandet T, Kaplan D, Llanwarne C, Munshi R, Novod S, Petrillo N, Roazen D, Ruano-Rubio V, Saltzman A, Schleicher M, Soto J, Tibbetts K, Tolonen C, Wade G, Talkowski ME, Consortium TGAD, Neale BM, Daly MJ, MacArthur DG. Variation across 141,456 human exomes and genomes reveals the spectrum of loss-of-function intolerance across human protein-coding genes. *bioRxiv*. January 2019:531210. doi:10.1101/531210.

5. Sherry ST, Ward MH, Kholodov M, Baker J, Phan L, Smigielski EM, Sirotkin K. dbSNP: the NCBI database of genetic variation. *Nucleic Acids Res*. 2001;29(1):308-311. doi:10.1093/nar/29.1.308.

6. Schwarz JM, Cooper DN, Schuelke M, Seelow D. MutationTaster2: mutation prediction for the deep-sequencing age. *Nat Methods*. 2014;11(4):361-362. doi:10.1038/nmeth.2890.

7. Rentzsch P, Witten D, Cooper GM, Shendure J, Kircher M. CADD: predicting the deleteriousness of variants throughout the human genome. *Nucleic Acids Res*. 2019;47(D1):D886-D894. doi:10.1093/nar/gky1016.

8. Webb B, Sali A. Comparative Protein Structure Modeling Using MODELLER. In: *Current Protocols in Bioinformatics*. Hoboken, NJ, USA: John Wiley & Sons, Inc.; 2016:5.6.1-5.6.37. doi:10.1002/cpbi.3.

9. Berman HM. The Protein Data Bank. *Nucleic Acids Res*. 2000;28(1):235-242. doi:10.1093/nar/28.1.235.

10. Chen VB, Arendall WB, Headd JJ, Keedy DA, Immormino RM, Kapral GJ, Murray LW, Richardson JS, Richardson DC. MolProbity : all-atom structure validation for macromolecular crystallography. *Acta Crystallogr Sect D Biol Crystallogr*. 2010;66(1):12-21. doi:10.1107/S0907444909042073.

11. Pettersen EF, Goddard TD, Huang CC, Couch GS, Greenblatt DM, Meng EC, Ferrin TE. UCSF Chimera?A visualization system for exploratory research and analysis. *J Comput Chem*. 2004;25(13):1605-1612. doi:10.1002/jcc.20084.

12. Humphrey W, Dalke A, Schulten K. VMD: Visual molecular dynamics. *J Mol Graph*. 1996;14(1):33-38. doi:10.1016/0263-7855(96)00018-5.

13. Schrodinger LLC. The PyMOL Molecular Graphics System, Version 1.8. 2015.

14. Case DA, Ben-Shalom IY, Brozell SR, Cerutti DS, Cheatham III TE, Cruzeiro VWD, Darden TA, Duke RE, Ghoreishi D, Gilson MK. AMBER 2018: San Francisco. 2018.
